# An Information Theoretic, Microfluidic-Based Single Cell Analysis Permits Identification of Subpopulations among Putatively Homogeneous Stem Cells

**DOI:** 10.1371/journal.pone.0021211

**Published:** 2011-06-22

**Authors:** Jason P. Glotzbach, Michael Januszyk, Ivan N. Vial, Victor W. Wong, Alexander Gelbard, Tomer Kalisky, Hariharan Thangarajah, Michael T. Longaker, Stephen R. Quake, Gilbert Chu, Geoffrey C. Gurtner

**Affiliations:** 1 Department of Surgery, Stanford University School of Medicine, Stanford, California, United States of America; 2 Department of Bioengineering, Stanford University School of Medicine, Stanford, California, United States of America; 3 Departments of Medicine and Biochemistry, Stanford University School of Medicine, Stanford, California, United States of America; Emory University School of Medicine, United States of America

## Abstract

An incomplete understanding of the nature of heterogeneity within stem cell populations remains a major impediment to the development of clinically effective cell-based therapies. Transcriptional events within a single cell are inherently stochastic and can produce tremendous variability, even among genetically identical cells. It remains unclear how mammalian cellular systems overcome this intrinsic noisiness of gene expression to produce consequential variations in function, and what impact this has on the biologic and clinical relevance of highly ‘purified’ cell subgroups. To address these questions, we have developed a novel method combining microfluidic-based single cell analysis and information theory to characterize and predict transcriptional programs across hundreds of individual cells. Using this technique, we demonstrate that multiple subpopulations exist within a well-studied and putatively homogeneous stem cell population, murine long-term hematopoietic stem cells (LT-HSCs). These subgroups are defined by nonrandom patterns that are distinguishable from noise and are consistent with known functional properties of these cells. We anticipate that this analytic framework can also be applied to other cell types to elucidate the relationship between transcriptional and phenotypic variation.

## Introduction

A fundamental question for both developmental biology and regenerative medicine is how a single cell can generate a complex organism containing cells with diverse patterns of gene expression. Several investigators have demonstrated that numerous stochastic transcriptional events conspire to produce variations in patterns of expression among individual genetically identical cells [1], [2], [3], [4], [5]. Yet, transcriptional patterns at the organism level appear to be distinctly non-random [6], [7], [8]. The mechanisms underlying transcriptional stochasticity have been studied widely in bacteria and yeast [3], [4], [5], [9], [10], but their role in generating the heterogeneity observed in mammalian stem cell populations remains unknown. Traditional methods of gene expression analysis necessitate examination of pooled mRNA from thousands of cells, resulting in an averaged picture of gene expression across an entire cell population. Recent studies have increasingly employed technologies for analyzing gene expression within individual cells [3], [4], [11], [12]. The significant variations in gene expression demonstrated across individual cells by these investigations have made it clear that this transcriptional heterogeneity must be addressed in order to adequately describe a cell population [13], [14]. However, the relationship between stochastic variations of gene expression within individual cells and heterogeneous transcriptional profiles across a population of cells remains poorly understood.

A commonly used approach to characterize the heterogeneity of a large complex cell population (such as a stem cell population) is to fractionate the cells using surface antigen expression profiles and cell sorting strategies such as fluorescence activated cell sorting (FACS). As sorting strategies become more sophisticated, distinct functional subgroups of cells emerge. One method to predict whether a cell subgroup still harbors *phenotypic* variation (*i.e.* is still heterogeneous), is to determine if it can be further broken down into subpopulations with meaningful *transcriptional* differences between them (Figure S1).

Bone marrow hematopoietic stem cells (HSCs) are an ideal system in which to explore the relationship between stochastic noise and meaningful variations in transcriptional profiles. In the bone marrow niche, cells exist as individual units, yet function collectively to create a complex hierarchical organ system (the blood) [15]. Each level of the canonical HSC lineage hierarchy has been defined, allowing prospective isolation of each cell type with a high degree of purity [16]. At the pinnacle of this hierarchy, long-term HSCs (LT-HSCs) exist as a putatively homogenous and largely quiescent population with the potential to generate all the cells of the hematopoietic system [16], [17], [18]. However, the homogeneity of this compartment has been recently been called into question by the work of Wilson *et al.*, which demonstrated that a tightly sorted LT-HSC population harbors significant functional heterogeneity with regard to cell cycling and stem cell capacity [19]. The molecular basis for this heterogeneity cannot be elucidated from pooled populations of cells, but requires single cell analysis [13], [20], [21].

**Figure 1:**
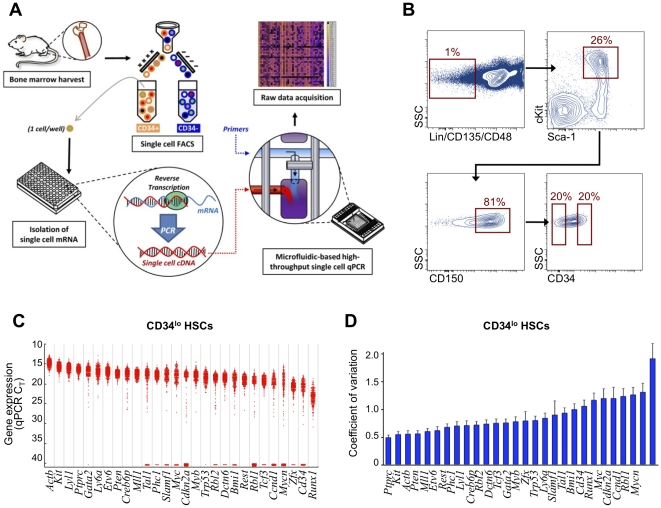
Single cell gene expression analysis demonstrates transcriptional variation in murine LT-HSCs. (**A**) Schematic of high throughput microfluidic chip-based single cell transcriptional analysis. A single cell is sorted by FACS into each well of a 96-well plate that has been preloaded with RT-PCR reagents (see methods for complete description). A low-cycle RT-PCR pre-amplification step creates cDNA for each gene target within each individual cell. Single cell cDNA is then loaded onto the microfluidics chip along with the primer-probe sets for each gene target. The BioMark machine performs qPCR for each cell across all 48 gene targets in parallel, resulting in 2,304 data points for each chip run. (**B**) FACS sorting parameters of two populations of HSCs isolated from primary murine bone marrow. All cells were LSK (Lin^neg^ Sca-1^+^ cKit^+^) CD48^–^ CD135^–^CD150^+^ and were sorted into two distinct populations based on CD34 expression (CD34^lo^ and CD34^hi^). SSC  =  side scatter. (**C**) Histogram presenting raw qPCR cycle threshold values for individual genes across 300 LT-HSCs. Each dot represents a single gene/cell qPCR reaction, with increased cycle threshold values corresponding to decreased mRNA content. Cycle threshold values of 40 were assigned to all reactions that failed to achieve detectable levels of amplification within 40 qPCR cycles. For convenience, genes that failed to amplify in the majority of cells have been omitted (see Figure S1 for complete dataset). (**D**) Single-gene coefficient of variance (COV) values for individual CD34^lo^ HSCs. Error bars represent standard deviations derived through bootstrapping over 100,000 iterations as previously described [50].

Microfluidic-based platforms are being increasingly utilized to interrogate gene expression on the single cell level [22], [23], [24], [25]. We and others have previously demonstrated that high-resolution single cell transcriptional analysis is efficient and reliable on a small scale using single cell FACS and multiplexed quantitative polymerase chain reactions (qPCR) within a chip-based microfluidic large-scale integration system [12], [23], [24], [26], [27], [28], [29]. Here, we apply this analytic method to allow more thorough interrogation of the heterogeneity present within the LT-HSC compartment at the single cell level using microfluidic-based single cell transcriptional analysis (Figure 1A). We apply a computational method employing principles of information theory to interpret the resulting single cell data. Using this approach, we demonstrate that nonrandom levels of transcriptional heterogeneity are present within this putatively homogenous stem cell population.

## Results

### HSC Cell Sorting

We reproduced the sorting strategy used previously to define LT-HSCs [19] to isolate 300 individual cells from the CD34^lo^ fraction of the LSK (lineage^neg^ Sca-1^+^ cKit^+^) CD48^-^ CD135^-^ CD150^+^ subset of primary murine bone marrow (Figure 1B). For each of the LT-HSCs, we measured the expression of 43 genes known to be highly relevant to hematopoiesis (Table S1) using a microfluidic-based method [29]. This work represents the largest study to date of gene expression in single cells from a purified murine hematopoietic stem cell population, both in terms of the number of cells and number of genes analyzed.

### Single-Cell Transcriptional Variability

As expected, we observed cell-to-cell variation in the expression of all genes (Figures 1C and S2). The expression patterns for many genes followed a relatively normal distribution (*e.g.*, the structural gene *Actb*, the hematopoietic surface antigen *Ptprc*, and the transcription factor *Runx1*). Such gene distributions exhibited a tendency for decreased relative transcriptional variation with increasing mRNA expression, consistent with prior observations [6], [30]. However, some genes (*e.g.*, the transcription factor *Tal1* and the cell cycle-related genes *Cdkn2a*, *Rbl1*, and *Ccnd1*) displayed markedly asymmetric transcriptional distributions. These variations may be the result of transcriptional bursts as reported by others [30], [31] or could arise from physiological factors, such as discontinuities across the cell cycle.

We have previously shown that the univariate coefficient of variance (COV) can be used to describe single gene variability (Equation 1) [26]. In the present study, the range of COV for individual genes (0.5 [*Ptprc*, also known as CD45] to 1.9 [*Mycn*]) (Figure 1D) was consistent with that previously reported for murine HSCs [12], [26]. If all variations in gene expression were completely independent, we could extend this analysis to construct a rudimentary index of population heterogeneity based on the multivariate generalization of COV over a given set of *n* genes (Equation 2) [32]. 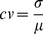
(1)

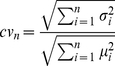
(2)Each σ_i_^2^ refers to a single gene variance, with µ_i_ representing its mean level of expression. The resulting dimensionless index (*cv_n_*) would provide a standardized, scale-invariant measure of dispersion. However, this simple metric fails to account for co-variations among genes, which are present throughout our transcriptional data. Thus a more comprehensive approach to evaluate heterogeneity within LT-HSCs is needed.

Prior theoretical work has attempted to determine whether cell-to-cell transcriptional variation arises from noise-generated fluctuations around a stable fixed point in a homogeneous population or whether variation arises from multiple eigenstates within a heterogeneous population [6], [7], [33]. Many of these efforts have focused on modeling transcriptional noise through the framework of statistical mechanics, in which system-wide gene expression is reduced to a master equation describing the evolution of gene-state probability distributions over time [6], [33]. These models have provided valuable insight into the mechanisms of the cellular transcriptional machinery, particularly for regulatory feedback systems near equilibrium that would attenuate noise in data such as ours. Most experimental studies investigating single cell gene expression and stochasticity have focused on the changes within an individual cell over time [3], [4], [10] or have addressed only a small number of genes [5], [11], [34]. Here, we measured transcription in 300 cells from a tightly sorted population at a single point in time.

### Establishing a Threshold for Transcriptional Homogeneity

An ideal test for homogeneity would compare the transcriptional distribution measured across a population to some fixed level of baseline noise. However, at present no consensus exists regarding the basal level of variability inherent to steady-state gene transcription, and we expect that the magnitude of this noise would (1) vary with absolute mRNA quantity (*i.e.*, not hold constant) and (2) depend upon the intrinsic biochemical properties of specific genes or gene classes [6]. Given the current limitations in measurement technology, such a dynamical systems approach to characterize baseline transcriptional heterogeneity becomes unwieldy for even very small numbers of genes, suggesting that an absolute threshold for homogeneity will be difficult to define.

Alternatively, one could apply traditional statistical methods to compare the variability observed across a given population against that of a “control” group (generally accepted to be phenotypically homogeneous, *e.g.* a clonal cell line), evaluated using an identical panel of genes. However, the multipotent nature of LT-HSCs is such that those genes which best characterize this population are not, to our knowledge, universally expressed across any other cell type. Further, the capacity of LT-HSCs for differentiation has precluded comparative evaluation of a clonal LT-HSC population. These inherent limitations are not unique to LT-HSCs, and may be relevant to the study of many rare cell populations.

These factors have motivated us to develop an approach using principles of information theory and statistical physics to test the hypothesis of relative transcriptional homogeneity. Information theory focuses on understanding and correcting for randomness or entropy within a dataset to allow quantification and interpretation of heterogeneous data, and work in statistical physics has generated methods for applying probability functions to inherently stochastic processes. In the absence of an acceptable external comparison, these methods permit us to utilize relationships derived from the variability within our data itself in order to provide insight into the dynamics of this complex system. This approach itself is not novel, and similar methods have been applied with great success to problems in signal processing and control theory [35], [36]; however, these techniques have only recently gained traction as tools to characterize biological systems [37], [38], [39].

**Figure 2:**
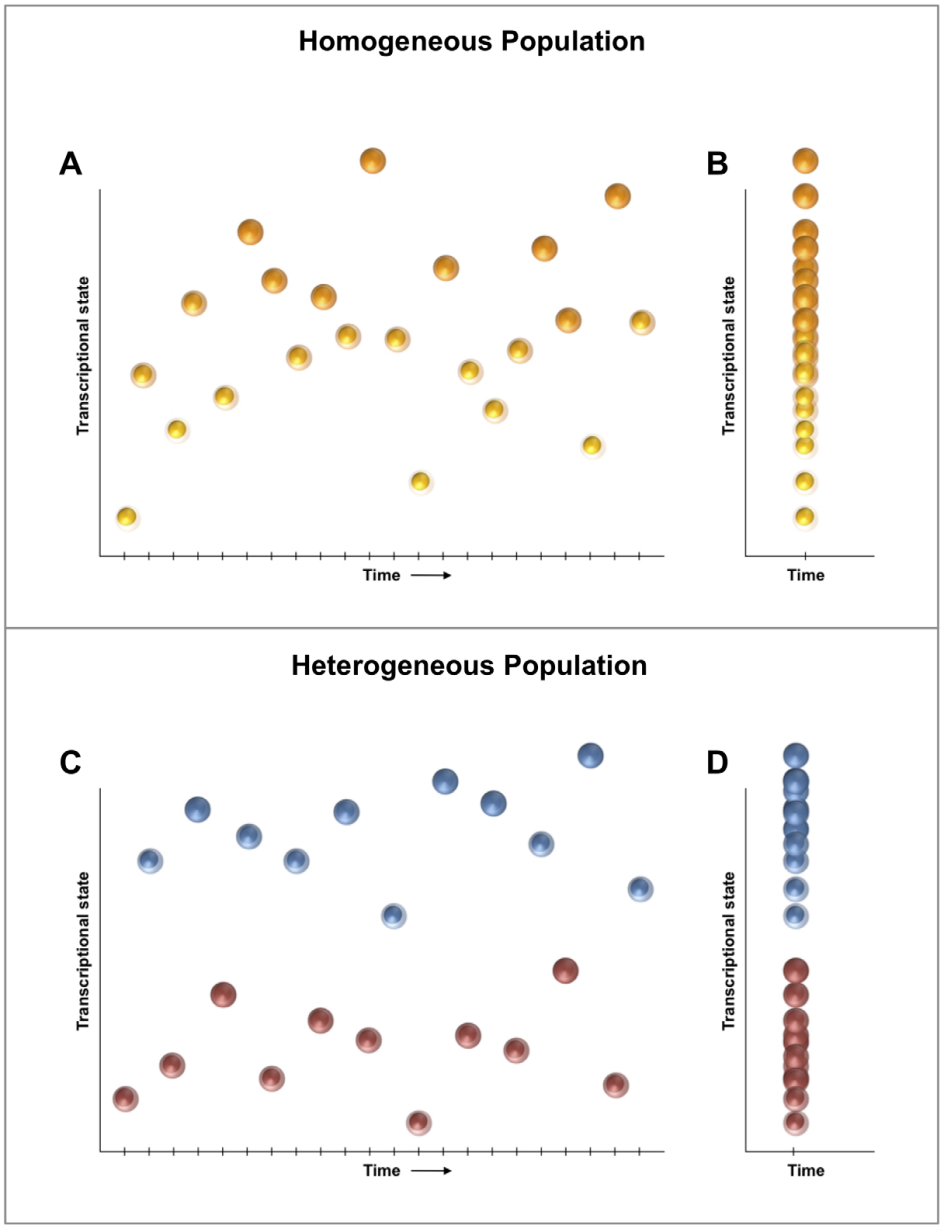
A transcriptional distribution-based model of population homogeneity. Given the noisiness inherent to transcription, an individual cell will exhibit a variable transcriptional signature if measured precisely over time (**A**). A cell population can be considered “homogeneous” if all individual cell transcriptomes are governed by identical steady-state probability functions (*i.e.* all cells are drawn from a single probability field). It follows that the transcriptional fingerprint of a homogeneous population measured at a single timepoint (**B**) should, through the transcriptional states of all individual cells, recapitulate the single distribution observed for any one cell measured across multiple time points (**A**). By contrast, if the distribution of individual cell transcriptomes from a population at a single timepoint (**D**) more closely reflect that of two (or more) independent probability functions (**C**), then the population may be designated as heterogeneous.

We stipulate that a given population (P_n_) of n cells (with transcriptomes T_1_, …T_n_) is “homogeneous” if all individual cell transcriptomes are governed by identical steady-state probability functions (*i.e.* all cells are drawn from a single probability field) (Figure 2A). It follows that the transcriptional fingerprint of a homogeneous population measured at a single timepoint should recapitulate this single distribution through the transcriptional states of all individual cells (Figure 2B). Thus, establishing the homogeneity of P_n_ is equivalent to demonstrating that no set of subpopulations P^’^_1_∪P^’^_2_∪ … ∪P^’^_s_  =  P_n_ exists for which the observed data (T_1_, … T_n_) are more likely to have arisen from their joint probability distribution than from P_n_ itself. Conversely, if a cell population represents two or more probability distributions, it can be considered heterogeneous (Figure 2C, D). We applied this paradigm to our multivariate system of gene expression in order to evaluate the heterogeneity of this highly purified population of LT-HSCs.

### Clustering Algorithm, Feature Selection, and Optimization

**Figure 3:**
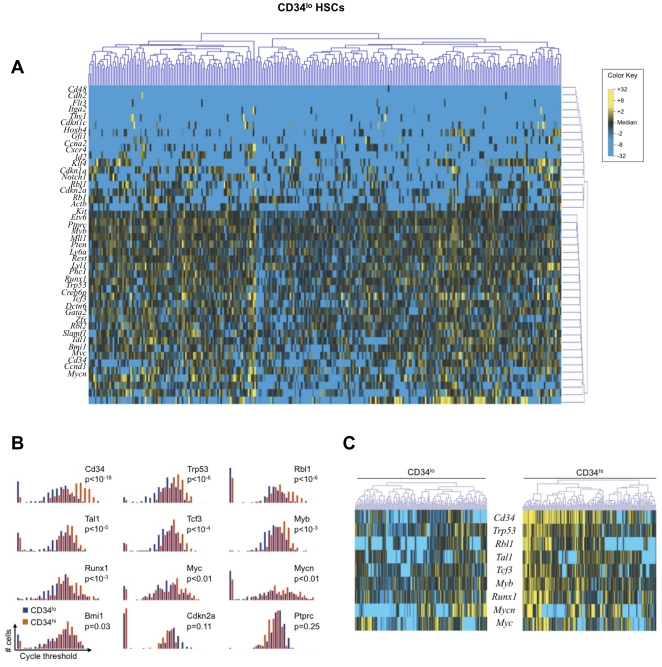
A multivariate, information-theoretic approach permits characterization of patterns in higher-order correlated gene expression. (A) Hierarchical clustering of simultaneous expression of 43 genes among 300 individual CD34^lo^ HSCs. Gene expression is presented as fold change from median on a color scale from yellow (high expression, 32-fold above median) to blue (low expression, 32-fold below median). (B) Differentially-expressed genes between CD34^lo^ and CD34^hi^ HSCs identified using non-parametric two sample Kolmogorov-Smirnov testing. Nine genes exhibit significantly different (*p* < 0.01 following Bonferroni correction for multiple comparisons) distributions of single cell expression between the two populations, illustrated here using median-centered histograms (bin size  =  0.5 qPCR cycle thresholds). (C) Comparison of CD34^lo^ and CD34^hi^ populations. Cells are clustered hierarchically based on a Kolmogorov-Smirnov-significant gene subset.

**Figure 4:**
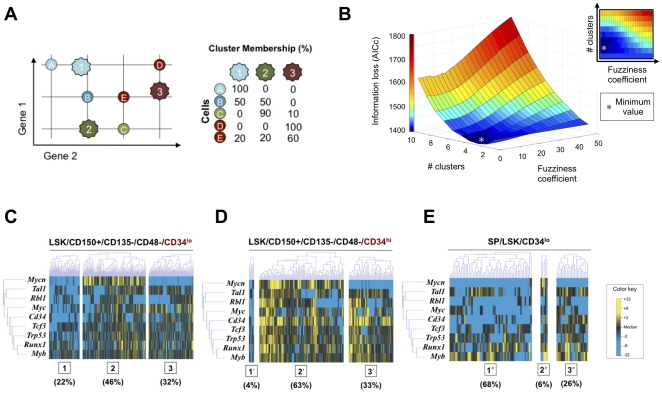
Optimized partitive modeling of LT-HSC single cell transcriptional data. (**A**) Individual cells are clustered within a hypothetical 2-gene space (represented by horizontal and vertical axes). Fuzzy c-means clustering allows shared membership of an individual cell within two or more clusters. Cluster centers (k1, k2, k3) are determined based on the similarities across all cells in the sample. A “fuzziness coefficient” modulates the degree to which partial membership is encouraged among clusters. (**B**) Iterative application of Akaike Information Criterion (with a second order correction for small sample sizes [44]) to determine optimal clustering parameters. An exhaustive approach was used to determine the information loss (z-axis) associated with different permutations of the number of clusters (y-axis) and the fuzziness coefficient (x-axis). The trough of the three dimensional plot (grey asterisk) represents the optimal set of clustering parameters for the given data set that will minimize theoretical information loss. (**C–E**) Fuzzy c-means clustering of HSC single cell transcriptional data using the optimal clustering parameters (3 clusters and a fuzziness coefficient of 1.05). Only the Kolmogorov Smirnov-significant genes (Figure 3B) are displayed for visual simplicity. Cluster centroids are determined based on partitioning of the CD34^lo^ cells and applied across the other two experimental groups. (**C**) CD34^lo^ HSCs are relatively evenly distributed across the three clusters. (**D**) CD34^hi^ HSCs demonstrate a substantially different distribution. Membership in cluster 1′ is limited to 4% of the cells and cluster 2′ membership is the most common. (**E**) Side population CD34^lo^ HSCs would be expected to be substantially enriched for HSC capacity and should resemble the CD34^lo^ HSCs. Membership in cluster 1′′ is significantly expanded, suggesting that cells in this subpopulation are characteristic of highly enriched LT-HSCs.

In order to determine whether LT-HSCs (Figure 3A) represent a homogeneous population or several discrete subpopulations, we applied a unifying procedure for model selection and multimodal inference based on the principles of information divergence, originally described by Kullback and Leibler [40]. In order to increase statistical efficiency, a subset of genes was selected whose transcriptional variation would most likely represent meaningful differences among cells. To accomplish this, we employed Kolmogorov-Smirnov statistics to compare CD34^lo^ cells against a population of otherwise identically sorted CD34^hi^ HSCs (Figure 1B), which have been shown to harbor a much lower stem cell capacity [19]. This identified a subset of nine genes with distributions of expression that were different between the two cell populations (*p* < 0.01 following Bonferroni correction for multiple comparisons) (Figure 3B, C) [19], [40], [41], [42], [43], [44]. Transcriptional data for all LT-HSCs were evaluated using a generalized fuzzy c-means clustering algorithm, which permits partial memberships via “soft partitions” representing overlap in probability distributions (Figure 4A) [43]. We then utilized an information metric, Akaike Information Criterion (AIC), to assess the “goodness of fit” for each of the resulting cluster configurations, optimizing the cluster parameters (*i.e.*, cluster number and fuzziness coefficient) in order to minimize information loss (Figure 4B). This permits robust, objective comparison of the single-cluster model against all permutations of multi-cluster alternatives [40], [44].

### HSC Cluster Membership

In the optimal partitive model, as determined by our method, CD34^lo^ HSCs distributed relatively evenly across three clusters, each with distinctive transcriptional fingerprints (Figure 4C). The number of clusters was found to be relatively stable against changes in cell selection, gene selection, gene number, and clustering algorithm (Figures S3, S4, S5). For these analyses, certain genes (*e.g.*, *Mycn* and *Cdkn2a*) consistently showed high expression associated with specific clusters, whereas other genes (for example, *Runx1* and *Myb*) exhibited stochastic variation among clusters (Figure 4C and Figures S3B-D). Thus our results suggest the presence of both stochastic and nonstochastic variations in gene expression.

We organized the CD34^hi^ HSCs around the cluster centroids generated through the clustering of CD34^lo^ HSCs, and observed a dramatically different distribution across these three clusters (Figure 4D). CD34^lo^ HSCs have been shown by others to contain a much larger subset of dormant LT-HSCs with a high stem cell capacity in comparison to CD34^hi^ HSCs [19]. Thus, it is possible that the different clusters identified by our analysis reflect subpopulations that account for the observed functional differences between these two HSC populations. To test this relationship, we utilized an alternate isolation protocol for LT-HSCs that yields a side population (SP) based on cellular Hoechst dye 33342 extrusion (Figure S6) [45], [46]. When organized around the same cluster centroids, 68% of the SP LSK CD34^lo^ cells were associated with cluster 1 (Figure 4E). Taken together, these findings suggest that LT-HSC capacity may exist within a subpopulation of cells that are largely absent from the CD34^hi^ population (cluster 1, Figure 4) and that additional surface marker sorting will be needed to isolate a homogeneous population of LT-HSCs.

## Discussion

Single cell analysis is essential to understand the heterogeneity within rare or complex cell populations (such as stem cells); however techniques for interpreting this fundamentally new type of data are still in their infancy. Recent technological advances have vastly increased the ability of qPCR to detect gene expression within a single cell [4], [27]. With these high-resolution measurements, it has become apparent that transcription is an inherently noisy process at the level of a single gene within an individual cell. In order for single cell transcriptional analysis to contribute to our understanding of cell biology, it must overcome the ambiguity created by this noisiness. To address this need, we have developed a novel application of microfluidic technology coupled with analytic principles from information theory that defines transcriptional signatures of individual cells and provides the capability to discriminate-on the single cell level-meaningful variation (signal) from background stochasticity (noise) in the transcriptomes of a heterogeneous cell population.

We designed our analytic approach to perform direct computation of correlations in cell/gene expression at the single cell level and identify groups of cells that exhibited similar patterns of higher-order correlated gene expression, similar to the way in which classical microarray cluster analysis identifies groups of genes that exhibit similar patterns across multiple tissue samples [47]. We elected to cluster these data using an adaptive fuzzy c-means algorithm, a well-established extension of traditional k-means clustering [43] that permits partial membership for each cell in multiple clusters. This method is well-suited to the temporal framework of our data, which is essentially a snapshot in time of a dynamic system, and was stable against small perturbations in our dataset, converging to the k-means solution (Figures S3 and S4) [48]. Although AIC itself is poorly suited to traditional null hypothesis testing, multiple methods have been developed to evaluate uncertainty in model selection [44]. Application of these information theoretic measures to examine differences among the canonical 1-cluster model, the “optimal” (three-cluster) model, and other cluster arrangements, supports the conclusion that these highly purified cells exist in distinct subpopulations rather than as one homogeneous population (Table S2).

We therefore establish the effectiveness and relevance of our large-scale computational method by demonstrating non-random, transcriptionally defined subpopulations that have not previously been described within the well-studied and putatively homogenous murine LT-HSC cell population. Our results demonstrate the feasibility of measuring gene expression in multiple individual cells from a stem cell population using single cell qPCR in a multiplexed array based on microfluidic large-scale integration technology [27]. Using this approach, we detected variations in gene expression profiles within a well-studied murine LT-HSC population that could not be accounted for by stochastic transcriptional noise alone. Specifically, we identified several transcriptionally defined subpopulations that were consistent with the known functional heterogeneity of LT-HSCs [19].

It is important to note that post-transcriptional factors such as mRNA translation or protein modification may serve to mitigate (or amplify) the impact of this heterogeneity [5], [10]. In addition, these results will have to be confirmed with empirical testing of the functional differences displayed by the HSC subpopulations we describe here. This will require the identification and application of new sorting parameters to prospectively isolate these subpopulations. Given the observed variations in gene expression, this search is warranted, as the development of new sorting parameters may permit further enrichment of hematopoietic stem cells. More broadly, these findings demonstrate the utility of such an approach to define the transcriptional organization of complex cell populations on a tissue and organ level. We believe that this approach may be applied both for systems biology research and, potentially, for quality control to accompany the development of novel stem cell-based therapies.

## Materials and Methods

### Ethics Statement

All vertebrate animal work described in this manuscript was conducted according to the Stanford University Administrative Panel on Laboratory Animal Care (Protocol #12080), which specifically approved this study.

### Animals and HSC Isolation

11-week-old male C57BL/6 mice were purchased from Jackson Laboratories (Bar Harbor, ME). All animal protocols were approved by the Administrative Panel on Laboratory Animal Care at the Stanford University School of Medicine. After euthanasia, femora and tibiae were harvested and the marrow cavities were flushed with 2% fetal bovine serum (FBS) solution. Marrow plugs were dissociated by trituration, filtered through a 70 µm cell strainer, and pelleted by centrifugation. The cell suspension was incubated with biotin-conjugated murine antibodies against lineage surface antigens (CD5, CD45R, CD11b, Gr-1, 7-4, Ter119). After washing, non-labeled cells were extracted from the cell suspension using anti-biotin paramagnetic Micro Beads and the MACS separation system (Miltenyi Biotec, Gladbach, Germany).

### Antibody Staining and FACS Sorting

The following monoclonal antibodies were used in the experiments: CD11b-PECy5 (M1/70; eBioscience, San Diego, CA), CD45R-PECy5 (RA3-6B2; eBioscience), Gr-1-PECy5 (RB6-8C5, eBioscience), CD8a-PECy5 (53-6.7; eBioscience), CD4-PECy5 (GK1.5; eBioscience), Ter119-PECy5 (TER-119; eBioscience), cKit-AF700 (ACK2; eBioscience), cKit-APC (2B8; BD Pharmingen, San Diego, CA), cKit-PE (ACK45; BD Pharmingen), Sca-1-APC (D7; BioLegend, San Diego, CA), Sca-1-FITC (E13-161.7; BD Pharmingen), Sca-1-eFluor605 (D7; eBioscience), CD150-PECy7 (TC15-12F12.2; BioLegend), CD34-Pacific Blue (RAM34; eBioscience), CD34-FITC (RAM34, BD Pharmingen), CD48-PECy5 (HM48-1; BioLegend), CD48-APC (HM48-1; BioLegend), CD135-PECy5 (A2F10; eBioscience). Concentrations were determined based on the manufacturers' recommendations. For Hoechst dye extrusion (side population) studies, cells were incubated with 5 µg/mL Hoechst 33342 (Sigma-Aldrich, St. Louis, MO) for 90 minutes at 37°C. Control groups were also incubated with 50 µM verapamil (Fisher Scientific, Chicago, IL).

Lineage-depleted and stained bone marrow cells were sorted using a BD FACSAria equipped with a robotic cloning arm (Becton Dickinson Biosciences, San Jose, CA). To maximize the fidelity of the single cell sort and exclude unwanted cells, we used restrictive gating based on size and complexity and performed doublet discrimination to exclude aggregated cells. We doubled-sorted the cells, first with high precision 4-way purity parameters (yield mask 0/32, purity mask 32/32), followed by a single cell sort using maximal precision parameters (yield mask 0/32, purity mask 32/32 and phase mask 16/32) in order to minimize sorting errors.

### Microfluidic Chip-Based Single Cell Analysis

Single cell transcriptional analysis was performed as previously described [23], [24], [29]. Single cells were sorted into each well of a 96-well plate preloaded with 10 µL of a master mix containing Tris-EDTA buffer (pH 7.0), Superscript III reverse transcriptase enzyme (Invitrogen, Carlsbad, CA), Cells Direct reaction mix (Invitrogen, Carlsbad, CA), target gene-specific TaqMan assay (primer/probe) sets (Applied Biosystems, Foster City, CA) (Table S1), and SUPERase-In RNAse inhibitor (Applied Biosystems, Foster City, CA). Exon-spanning primers were used where possible to avoid amplification of genomic background. Cells were lysed and reverse transcription was performed (20 minutes at 50°C, 2 minutes at 95°C), followed by a gene target-specific 22-cycle pre-amplification (denature at 95°C for 15 minutes, anneal at 60°C for 4 minutes, each cycle). Resultant single cell cDNA was mixed with sample loading agent (Fluidigm, South San Francisco, CA) and Universal PCR Master Mix (Applied Biosystems, Foster City, CA) and loaded into 48.48 Dynamic Array chips (Fluidigm, South San Francisco, CA) along with TaqMan assays (Table S1) and assay loading agent according to the manufacturer's instructions (Fluidigm, South San Francisco, CA). Products were analyzed on the BioMark reader system (Fluidigm, South San Francisco, CA) using a hot start protocol to minimize primer-dimer formation, 30 quantitative PCR cycles were performed.

### Statistical Analysis

We utilized a well-established metric for comparison of empirical distributions, the two-sample Kolmogorov-Smirnov (K-S) test, to identify genes whose expression patterns differed significantly between population pairs (Figure 3B) using a strict cutoff of p<0.01 following Bonferroni correction for multiple samples. Expression data from all chips were normalized relative to the median expression for each gene in the pooled sample and converted to base 2 logarithms. Absolute bounds of +/− 5 cycle thresholds (corresponding to 32-fold increases/decreases in expression) were set, and zero-expressers were assigned to this floor.

In order to detect overlapping patterns within the single cell transcriptional data, we employed an adaptive fuzzy c-means clustering algorithm using a standard Euclidean distance metric. Each cell was assigned partial membership to each cluster as dictated by similarities in expression profiles. We employed an exhaustive optimization scheme using Akaike Information Criterion (AIC) with a second order correction for small sample sizes [44] to evaluate all possible combinations of cluster number and fuzziness coefficient, and selected parameters that minimized the theoretical “information loss” over our data [49]. Optimally partitioned clusters were then sub-grouped using hierarchical clustering in order to facilitate visualization of data patterning within and across these clusters. Figure S7 provides an overview of this process for a hypothetical set of single cell transcriptional data.

## Supporting Information

Figure S1 **Conceptual framework of transcriptional heterogeneity within a tightly sorted population of cells.** There likely exist several metastable and interconvertible transcriptional states of cells that combine to create a functionally heterogeneous population. Using precise single cell analysis, it is possible to determine whether the larger population of cells can be further subdivided into subpopulations that are different from each other, despite harboring a significant amount of stochastic variation within each subpopulation.
(TIFF)

Figure S2 **Histogram presenting raw qPCR cycle threshold values for each gene across all 300 LT-HSCs.** Individual dots represent single gene/cell qPCR reactions, with increased cycle threshold values corresponding to decreased mRNA content. Cycle threshold values of 40 were assigned to all reactions that failed to achieve detectable levels of amplification within 40 qPCR cycles.
(TIFF)

Figure S3 **Evaluation of cluster stability.** We evaluated the stability of our cluster-based approach with respect to changes in parameterization and dataset composition. (**A**) Bootstrapping was employed to evaluate 10,000 randomly selected subsets (70% [210 cells]) of our LT-HSC data. The AIC-optimal number of clusters varied from 2 to 4 across all iterations (mean  =  2.87; std. dev.  =  0.52), with an optimal model of 3 clusters selected in 71.8% of all permutations. Mean AIC values for each number of clusters (solid line) are depicted, with dashed lines delimiting one standard deviation. (**B**) We repeated our analysis using an alternate method for gene selection, choosing the nine genes with highest coefficients of variation (Figure 2D). The AIC-optimal model again consisted of three clusters, similar but not identical to those chosen with the earlier method. (**C–D**) Repeat analyses using the 8 (C) or 10 (D) genes with highest coefficients of variation, resulting in similar AIC-optimal models. (**E**) Information loss as a function of cluster number for the data in B–D (solid lines), compared with that from Fig. 4A (dashed line). (**F**) Information loss as a function of cluster number using gene selection based on Kolmogorov-Smirnov significance (Figure 4B).
(TIFF)

Figure S4 **Robustness analysis with respect to clustering technique.** Having demonstrated the stability of our approach with respect to changes in data and parameterization, we evaluated whether our findings could be artifacts of the approach itself. As no true precedent exists for data analysis of this type, we re-examined our data using the most simple form of partitional analysis (k-means clustering), in conjunction with a supervised classification method well-suited for clustering high-dimensional data without the need for feature selection (*i.e.*, a gene subset) to reduce the number of free parameters [48]. (**A**) Gene expression data for all 300 LT-HSCs were evaluated using a generic k-means algorithm, and the prediction strength of each k (number of clusters) calculated using five-fold cross-validation over 100 iterations as previously described. Cluster validation was achieved by maximizing the fidelity of pair-wise co-memberships of cells within clusters across repeated sub-samplings. The appropriate number of clusters was determined by the largest k whose prediction strength exceeds a certain threshold (typically set at 0.8) [48]. (**B**) Optimal partitioning of LT-HSCs using k-means with k = 3 clusters as determined above.
(TIFF)

Figure S5 **Robustness analysis with respect to distance metric.** In order to verify that our clustering results were not contingent upon any one specific measure of distance (i.e., the transcription-based assessment of divergence between two cells), we evaluated whether alternate metrics would produce significantly different partitioning schemes. (**A**) Euclidean distance was employed as the default measure throughout all clustering computations performed in this manuscript, resulting in the 3-cluster partition described in Figure 5C. (**B–** **C**) We repeated this central analysis using the Manhattan (or “city block”) distance measure (**B**), as well as the generalized Minkowski distance with order p = 3 (**C**). Grossly similar cluster configurations were achieved in both instances, suggesting that this arrangement is not an artifact attributable to any one metric.
(TIFF)

Figure S6 **Isolation of LT-HSC by the side population method.** (**A**) FACS plots of LT-HSC isolation using Hoechst dye extrusion (side population method), with three side population subfractions delineated (R1-R3), as previously reported [45]. Side population “tip” cells (R1) were isolated from lineage cell-depleted murine bone marrow cells and further sorted for lineage negative, cKit positive, Sca-1 positive, CD34^lo^ cells using identical gates to those presented in Figure 1B. (**B**) Abrogation of side population cells after incubation with verapamil in addition to Hoechst 33342.
(TIFF)

Figure S7 **Computational analysis schematic.** Gene expression data from multiple chips are pooled and blinded (top-left). Median-based normalization is applied gene-wise to ensure equal weighting for each gene (top-right). Fuzzy c-means clustering is used to group cells with similar expression profiles, and parameterization achieved through iterative application of Akaike Information Criterion (AIC) (bottom-left). Following cluster optimization, cells from within each group are arrayed according to hierarchical clustering (bottom-right).
(TIFF)

Table S1 **TaqMan assays used to interrogate gene expression within murine HSCs.** All assays were obtained from Applied Biosystems (Foster City, CA).
(TIFF)

Table S2 **Likelihood inference and model selection uncertainty.** “AIC differences” estimate the relative expected Kullback-Leibler differences between the true (underlying) distribution and that represented by the *i*-th model. The predicted best model (in this case, 3 clusters) will have an AIC difference of 0. Akaike weights correspond to the weight of evidence in favor of a given model being the actual best model (of those evaluated) for the dataset. Evidence ratios permit comparison of the relative likelihood of two models (in terms of Kullback-Leibler information). Here all evidence ratios are evaluated against the estimated best model (3 clusters) using the optimal fuzziness coefficient for each configuration.
(TIFF)
