# Supplementary figures and images for: An Information Theoretic, Microfluidic-Based Single Cell Analysis Permits Identification of Subpopulations among Putatively Homogeneous Stem Cells
Source: PLoS One. 2011 Jun 22;6(6):e21211. doi: 10.1371/journal.pone.0021211 (PMC3120839; doi:10.1371/journal.pone.0021211)

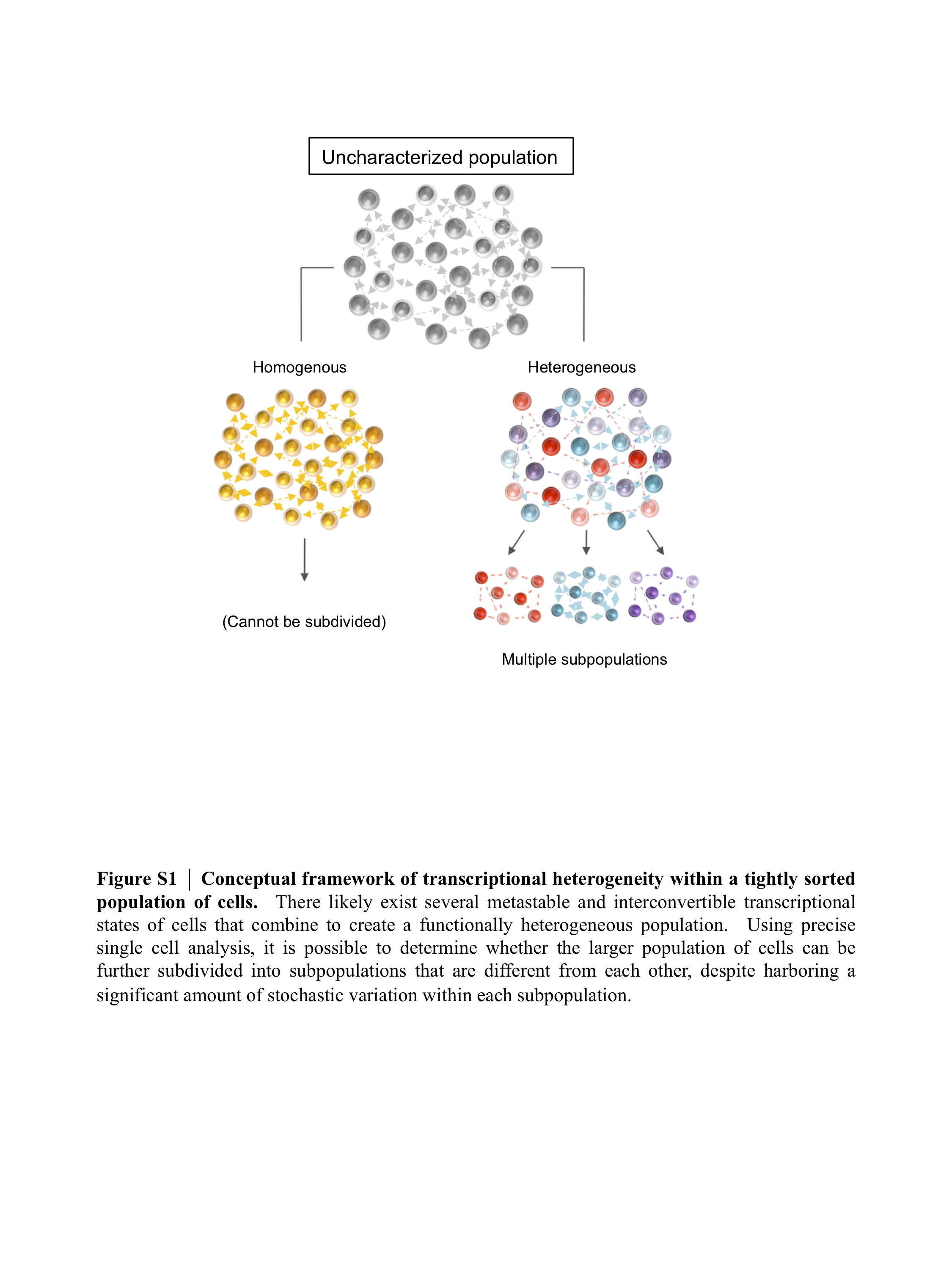

Supplement: Figure S1 — Conceptual framework of transcriptional heterogeneity within a tightly sorted population of cells. There likely exist several metastable and interconvertible transcriptional states of cells that combine to create a functionally heterogeneous population. Using precise single cell analysis, it is possible to determine whether the larger population of cells can be further subdivided into subpopulations that are different from each other, despite harboring a significant amount of stochastic variation within each subpopulation. (TIFF) [file pone.0021211.s001.tif]

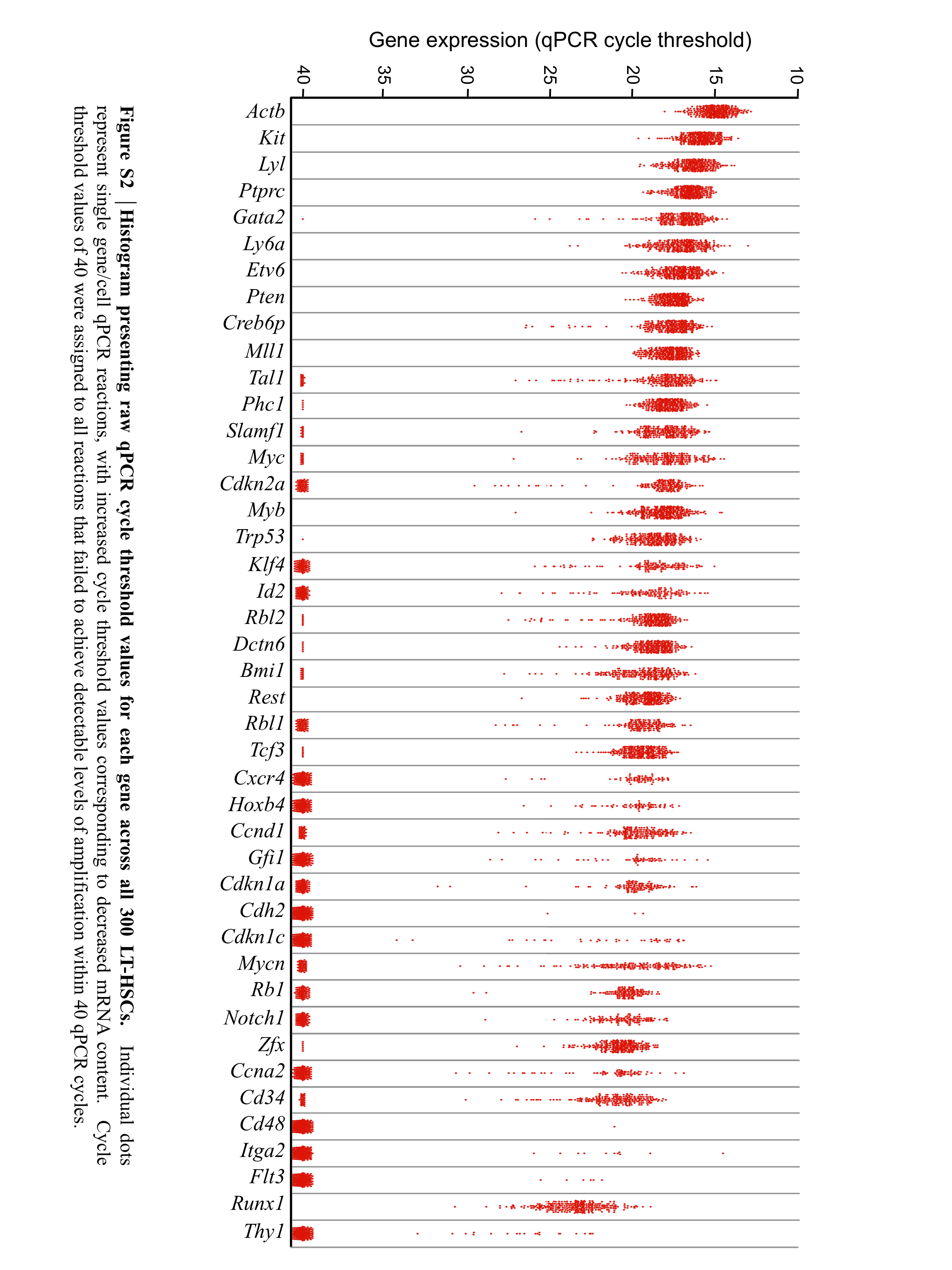

Supplement: Figure S2 — Histogram presenting raw qPCR cycle threshold values for each gene across all 300 LT-HSCs. Individual dots represent single gene/cell qPCR reactions, with increased cycle threshold values corresponding to decreased mRNA content. Cycle threshold values of 40 were assigned to all reactions that failed to achieve detectable levels of amplification within 40 qPCR cycles. (TIFF) [file pone.0021211.s002.tif]

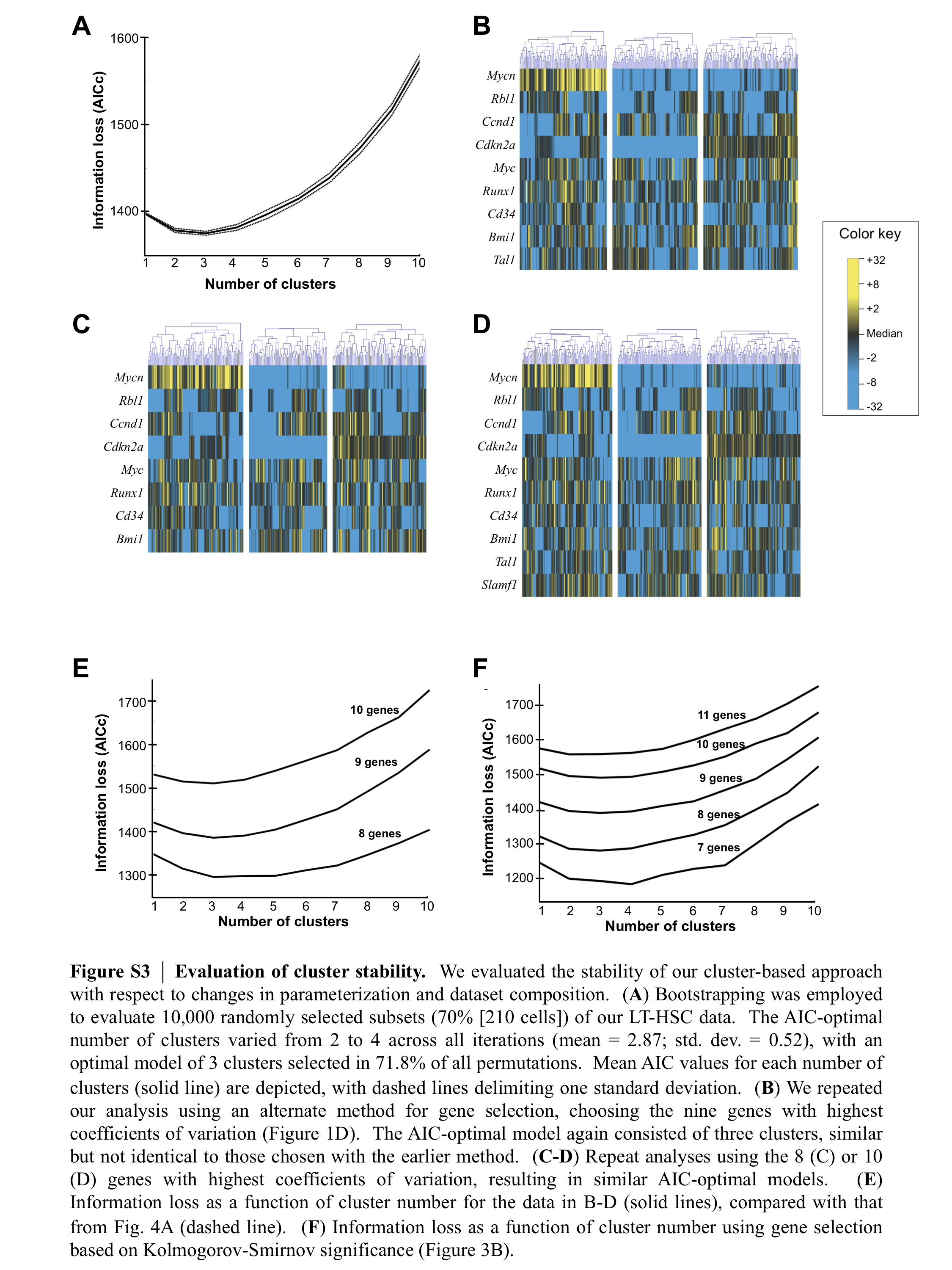

Supplement: Figure S3 — Evaluation of cluster stability. We evaluated the stability of our cluster-based approach with respect to changes in parameterization and dataset composition. (A) Bootstrapping was employed to evaluate 10,000 randomly selected subsets (70% [210 cells]) of our LT-HSC data. The AIC-optimal number of clusters varied from 2 to 4 across all iterations (mean = 2.87; std. dev. = 0.52), with an optimal model of 3 clusters selected in 71.8% of all permutations. Mean AIC values for each number of clusters (solid line) are depicted, with dashed lines delimiting one standard deviation. (B) We repeated our analysis using an alternate method for gene selection, choosing the nine genes with highest coefficients of variation (Figure 2D). The AIC-optimal model again consisted of three clusters, similar but not identical to those chosen with the earlier method. (C–D) Repeat analyses using the 8 (C) or 10 (D) genes with highest coefficients of variation, resulting in similar AIC-optimal models. (E) Information loss as a function of cluster number for the data in B–D (solid lines), compared with that from Fig. 4A (dashed line). (F) Information loss as a function of cluster number using gene selection based on Kolmogorov-Smirnov significance (Figure 4B). (TIFF) [file pone.0021211.s003.tif]

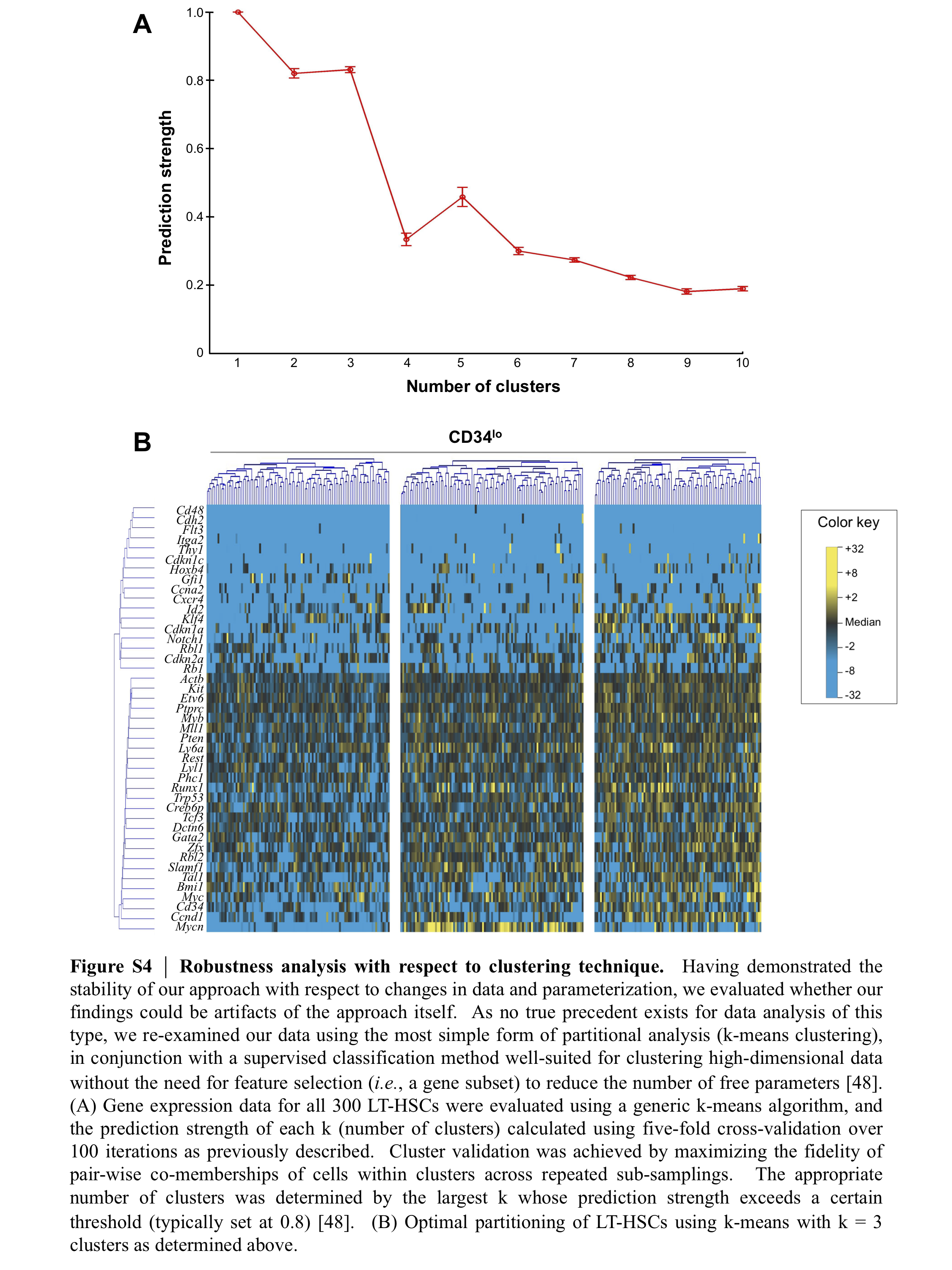

Supplement: Figure S4 — Robustness analysis with respect to clustering technique. Having demonstrated the stability of our approach with respect to changes in data and parameterization, we evaluated whether our findings could be artifacts of the approach itself. As no true precedent exists for data analysis of this type, we re-examined our data using the most simple form of partitional analysis (k-means clustering), in conjunction with a supervised classification method well-suited for clustering high-dimensional data without the need for feature selection (i.e., a gene subset) to reduce the number of free parameters [48]. (A) Gene expression data for all 300 LT-HSCs were evaluated using a generic k-means algorithm, and the prediction strength of each k (number of clusters) calculated using five-fold cross-validation over 100 iterations as previously described. Cluster validation was achieved by maximizing the fidelity of pair-wise co-memberships of cells within clusters across repeated sub-samplings. The appropriate number of clusters was determined by the largest k whose prediction strength exceeds a certain threshold (typically set at 0.8) [48]. (B) Optimal partitioning of LT-HSCs using k-means with k = 3 clusters as determined above. (TIFF) [file pone.0021211.s004.tif]

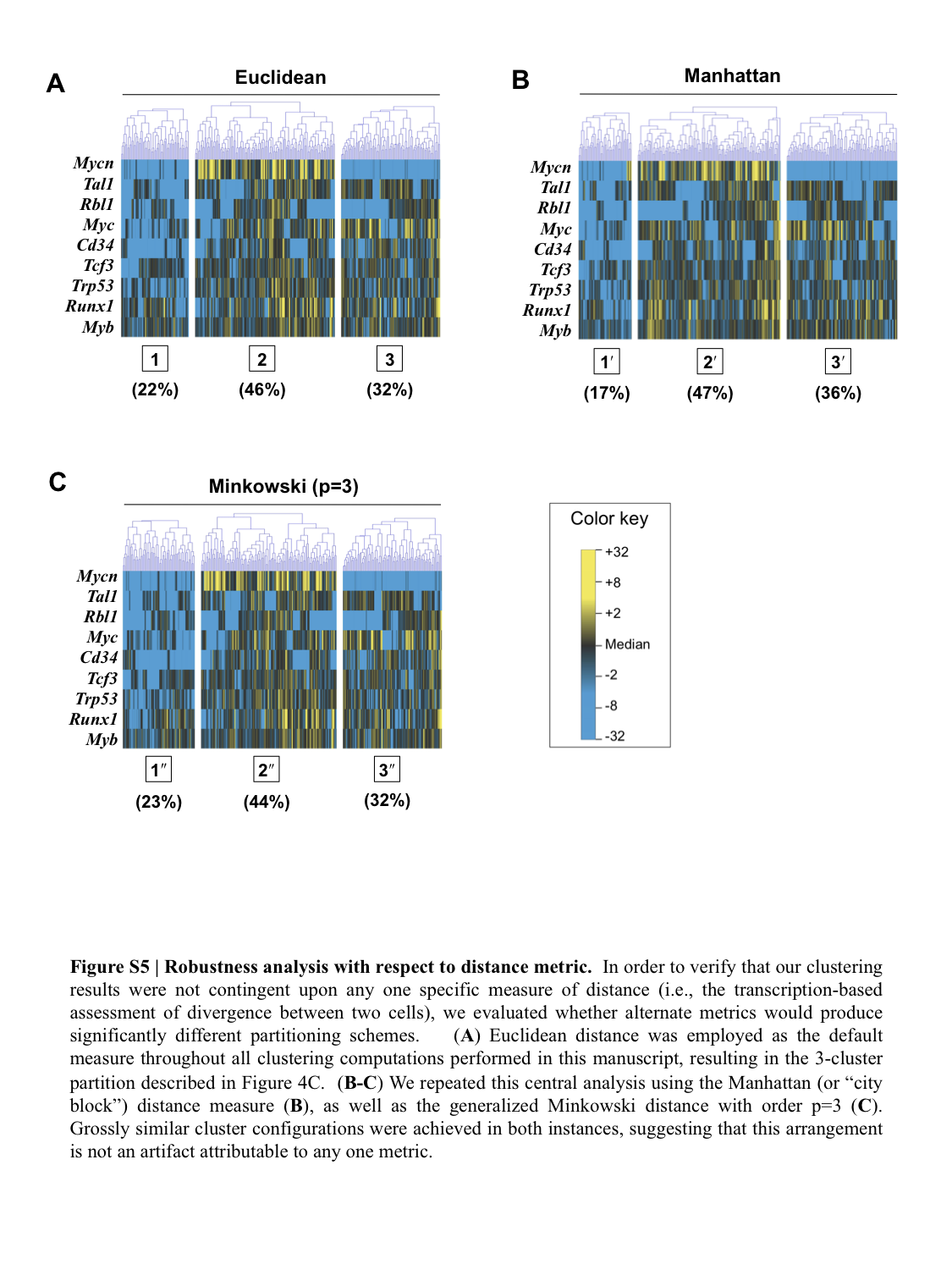

Supplement: Figure S5 — Robustness analysis with respect to distance metric. In order to verify that our clustering results were not contingent upon any one specific measure of distance (i.e., the transcription-based assessment of divergence between two cells), we evaluated whether alternate metrics would produce significantly different partitioning schemes. (A) Euclidean distance was employed as the default measure throughout all clustering computations performed in this manuscript, resulting in the 3-cluster partition described in Figure 5C. (B– C) We repeated this central analysis using the Manhattan (or “city block”) distance measure (B), as well as the generalized Minkowski distance with order p = 3 (C). Grossly similar cluster configurations were achieved in both instances, suggesting that this arrangement is not an artifact attributable to any one metric. (TIFF) [file pone.0021211.s005.tif]

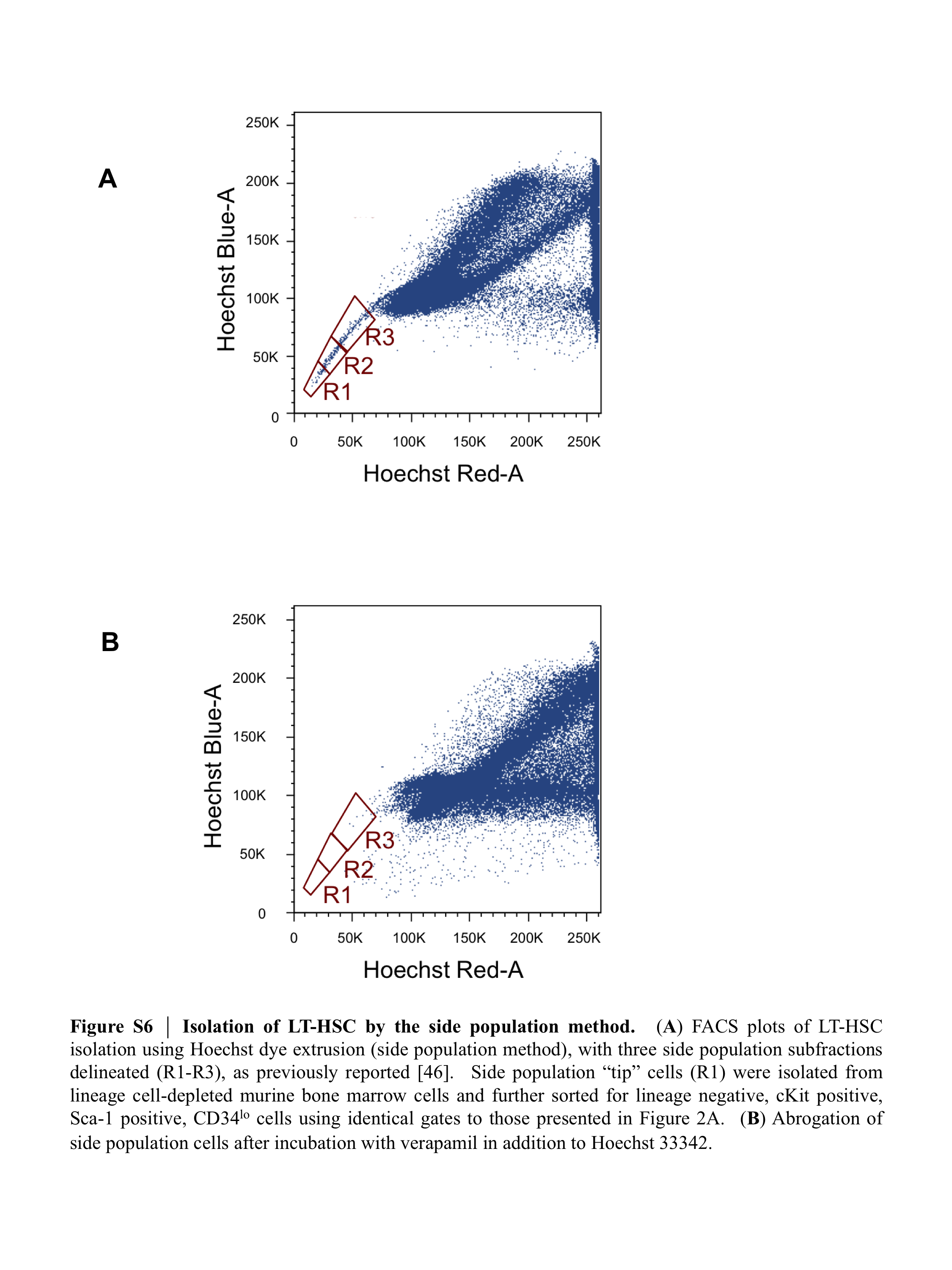

Supplement: Figure S6 — Isolation of LT-HSC by the side population method. (A) FACS plots of LT-HSC isolation using Hoechst dye extrusion (side population method), with three side population subfractions delineated (R1-R3), as previously reported [45]. Side population “tip” cells (R1) were isolated from lineage cell-depleted murine bone marrow cells and further sorted for lineage negative, cKit positive, Sca-1 positive, CD34lo cells using identical gates to those presented in Figure 1B. (B) Abrogation of side population cells after incubation with verapamil in addition to Hoechst 33342. (TIFF) [file pone.0021211.s006.tif]

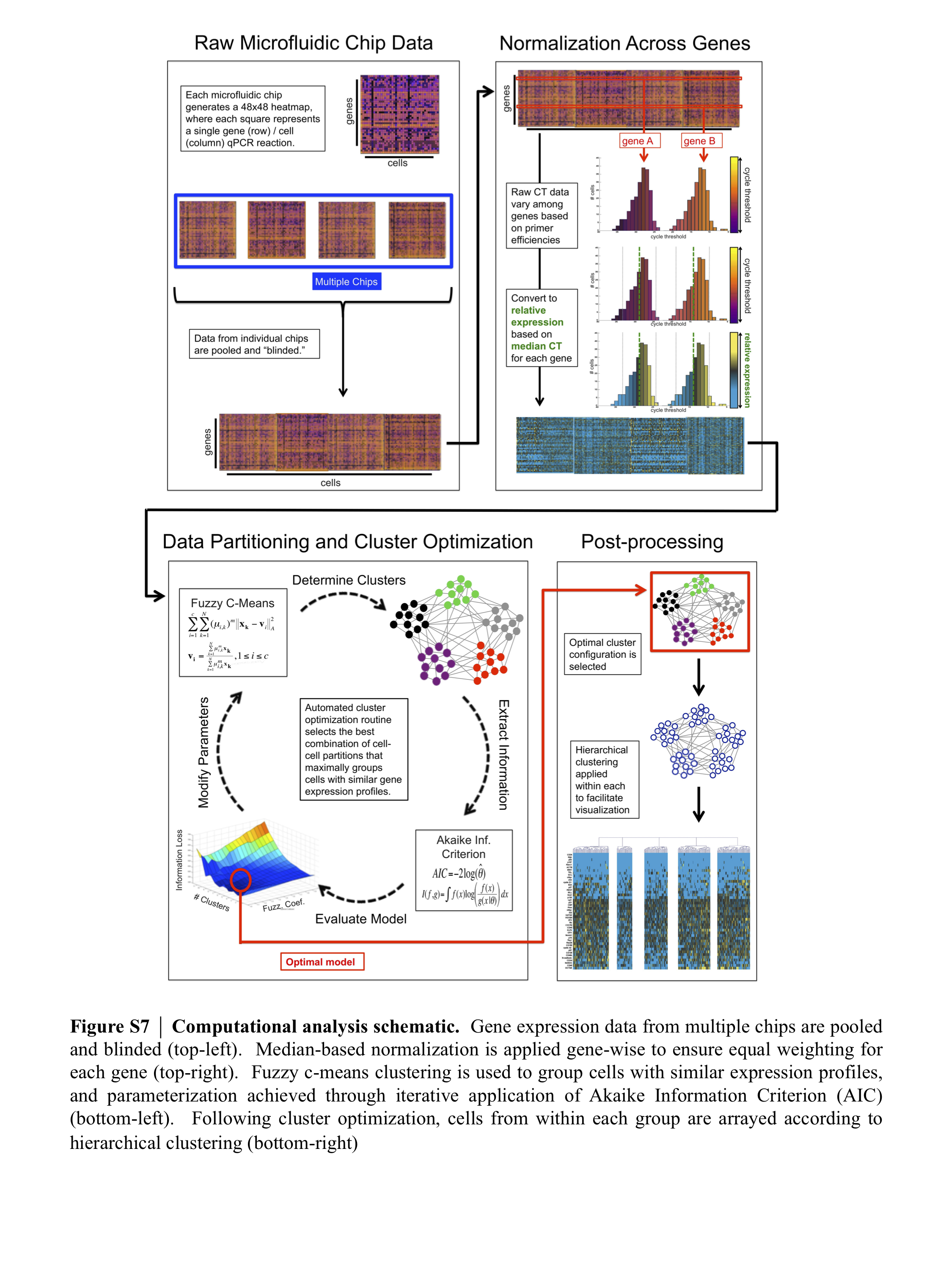

Supplement: Figure S7 — Computational analysis schematic. Gene expression data from multiple chips are pooled and blinded (top-left). Median-based normalization is applied gene-wise to ensure equal weighting for each gene (top-right). Fuzzy c-means clustering is used to group cells with similar expression profiles, and parameterization achieved through iterative application of Akaike Information Criterion (AIC) (bottom-left). Following cluster optimization, cells from within each group are arrayed according to hierarchical clustering (bottom-right). (TIFF) [file pone.0021211.s007.tif]

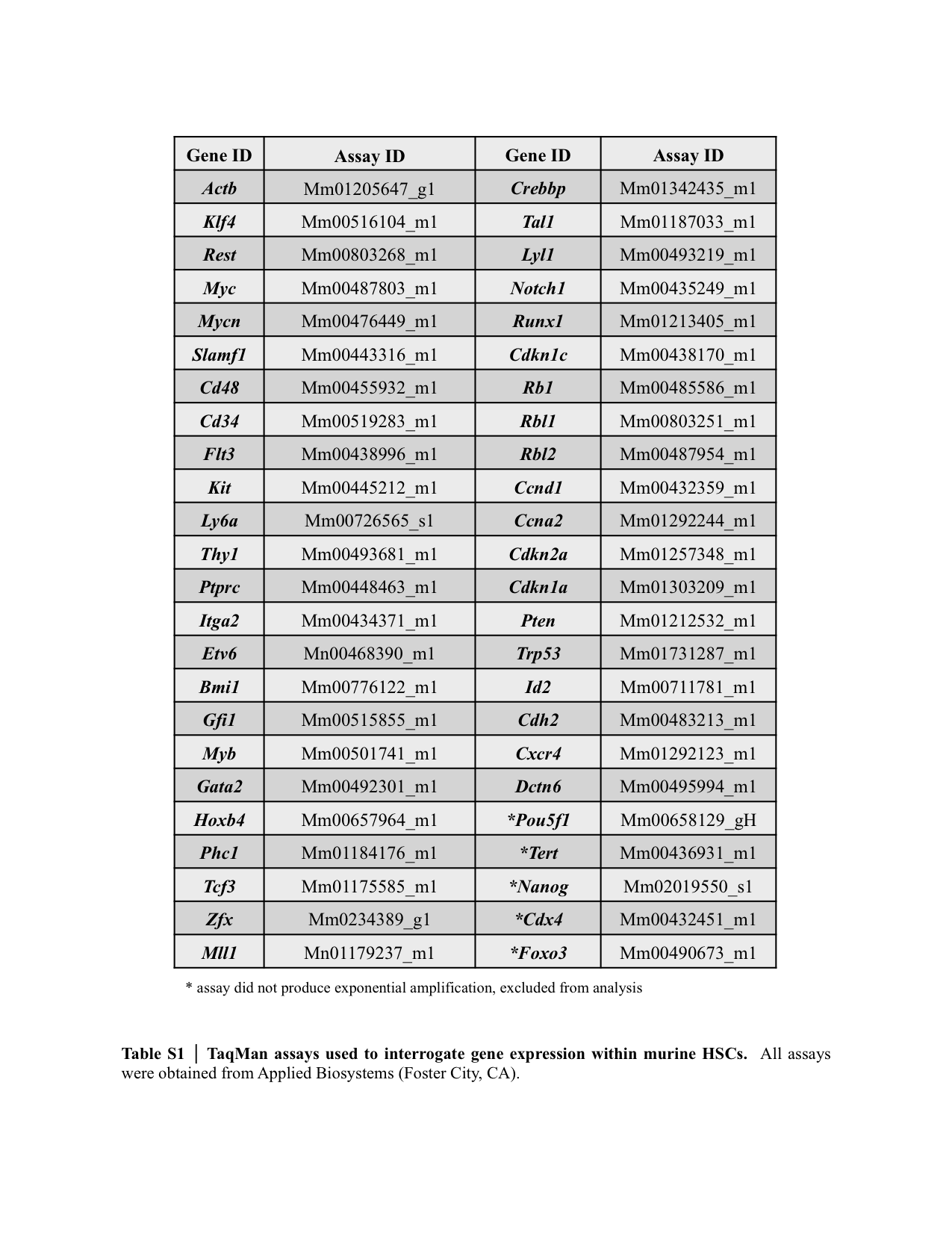

Supplement: Table S1 — TaqMan assays used to interrogate gene expression within murine HSCs. All assays were obtained from Applied Biosystems (Foster City, CA). (TIFF) [file pone.0021211.s008.tif]

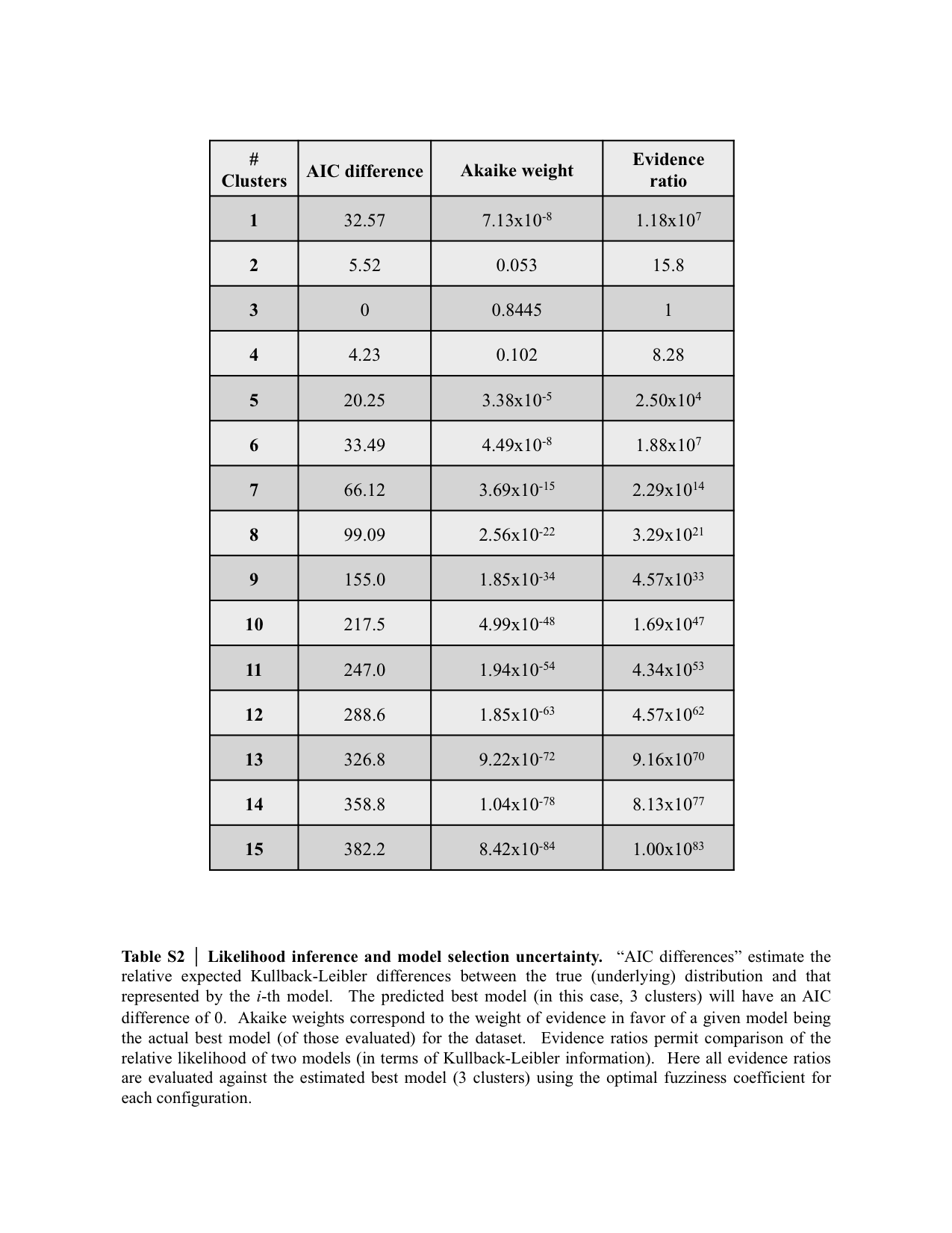

Supplement: Table S2 — Likelihood inference and model selection uncertainty. “AIC differences” estimate the relative expected Kullback-Leibler differences between the true (underlying) distribution and that represented by the i-th model. The predicted best model (in this case, 3 clusters) will have an AIC difference of 0. Akaike weights correspond to the weight of evidence in favor of a given model being the actual best model (of those evaluated) for the dataset. Evidence ratios permit comparison of the relative likelihood of two models (in terms of Kullback-Leibler information). Here all evidence ratios are evaluated against the estimated best model (3 clusters) using the optimal fuzziness coefficient for each configuration. (TIFF) [file pone.0021211.s009.tif]
